# Individual Differences in Attentional Breadth Changes Over Time: An Event-Related Potential Investigation

**DOI:** 10.3389/fpsyg.2021.605250

**Published:** 2021-03-23

**Authors:** Brent Pitchford, Karen M. Arnell

**Affiliations:** Department of Psychology, Brock University, St. Catharines, ON, Canada

**Keywords:** attentional breadth, event-related potentials, individual differences, attention, P3, N1

## Abstract

Event-related potentials (ERPs) to hierarchical stimuli have been compared for global/local target trials, but the pattern of results across studies is mixed with respect to understanding how ERPs differ with local and global bias. There are reliable interindividual differences in attentional breadth biases. This study addresses two questions. Can these interindividual differences in attentional breadth be predicted by interindividual ERP differences to hierarchical stimuli? Can attentional breadth changes over time within participants (i.e., intraindividual differences) be predicted by ERPs changes over time when viewing hierarchical stimuli? Here, we estimated attentional breadth and isolated ERPs in response to Navon letter stimuli presented at two time points. We found that interindividual differences in ERPs at Time 1 did not predict attentional breadth differences across individuals at Time 1. However, individual differences in changes to P1, N1, and P3 ERPs to hierarchical stimuli from Time 1 to Time 2 were associated with individual differences in changes in attentional breadth from Time 1 to Time 2. These results suggest that attentional breadth changes within individuals over time are reflected in changes in ERP responses to hierarchical stimuli such that smaller N1s and larger P3s accompany a shift to processing the newly prioritized level, suggesting that the preferred level required less perceptual processing and elicited more attention.

## Introduction

**Figure 1:**
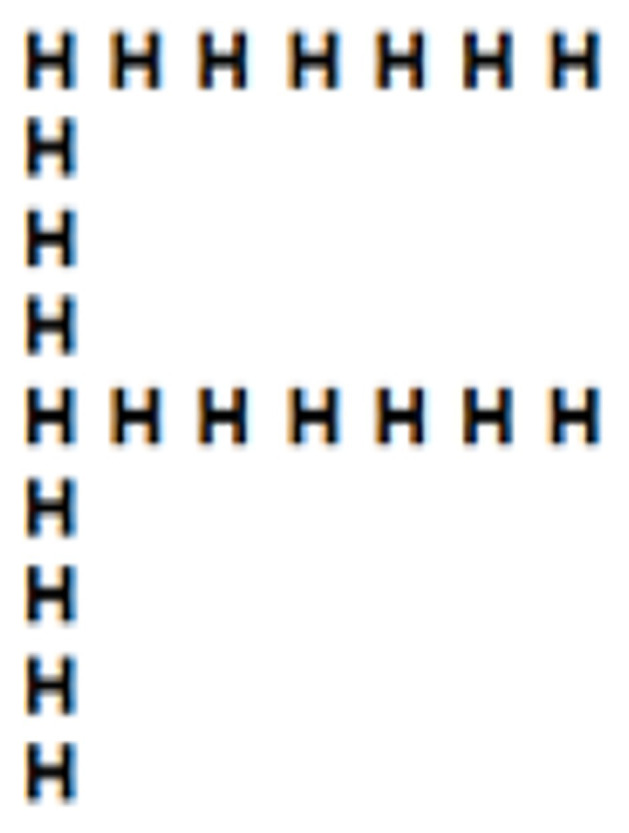
Example of an incongruent Navon letter stimulus (e.g., Navon, 1977).

Visual attention can be allocated to the entirety of a visual stimulus, or to its smaller details. Hierarchical Navon (1977) letters, such as the one presented in Figure 1, can be perceived at the global level as a large F, or at the local level as multiple smaller H’s. Stimuli such as these are often used to measure whether individuals are more attuned to the bigger picture (forest) or to the smaller details (the trees). One way to measure attentional breadth with such stimuli is to present only incongruent stimuli where one of two target letters (e.g., T or H) appears randomly at one level, while the other level contains one of two non-target letters (e.g., F or L). Individuals who are faster to report the identity of the target letter when it is presented in the global level relative to the local level would be said to show a global bias, whereas those that are faster to report the target letter when it is presented in the local level relative to the global level would be said to show a local bias (e.g., Gable and Harmon-Jones, 2008, 2011; Harmon-Jones and Gable, 2009; von Mühlenen et al., 2018; Pitchford and Arnell, 2019a,b). Greater attentional breadth is defined as a greater attentional focus on the global level suggesting that visual attention is more broad and diffuse, whereas greater attentional focus on the local level suggests that visual attention is more narrowed and attentional breadth is decreased.

Navon (1977) asked participants to report the letter present at the global level or at the local level using hierarchical letters that were either congruent (e.g., a large “T” made of smaller T’s) or incongruent (e.g., a large “F” made of smaller T’s). Results showed that the identification of local targets is impaired by incongruent information at the global level, but that identification of global targets is not as influenced by the congruence of the local information. Based on these results, Navon (1977) proposed the global precedence theory suggesting that there is a global advantage in that the global level information is processed faster than the local level. Indeed, many studies have found decreased reaction times (RTs) for global stimuli relative to local stimuli, and that the identification of local targets is impaired by incongruent information at the global level, but that identification of global targets is not as influenced by the congruence of the local information (Kimchi, 1992; Proverbio et al., 1998; Beaucousin et al., 2013; Dale and Arnell, 2013). However, the advantage for global stimuli can be attenuated or even reversed to an advantage for local stimuli if stimulus parameters are sufficiently manipulated (Shedden and Reid, 2001; Navon, 2003). Factors that have been previously shown to influence global/local processing include the visual angle (Kinchla and Wolfe, 1979), time of exposure (Paquet and Merikle, 1984), the aspect ratio, number, and relative density of the local elements (Kimchi and Palmer, 1982; Kimchi, 1992), and the hemisphere of presentation (Sergent, 1982; Kimchi and Merhav, 1991).

For decades, event-related electrophysiological brain potentials (ERPs) taken in response to hierarchical stimuli have allowed researchers to examine neural correlates of global and local processing (Proverbio et al., 1998; Yamaguchi et al., 2000; Malinowski et al., 2002; Beaucousin et al., 2013; Leek et al., 2016). Modulation of both early and later ERPs have been reported in selective attention tasks where individuals are instructed to report the identity of the letter at one level (e.g., global) in one block and then the letter identity of the other level (e.g., local) in another block (Han et al., 1997, 1999, 2000; Heinze et al., 1998; Proverbio et al., 1998; Evans et al., 2000; Yamaguchi et al., 2000; Beaucousin et al., 2011, 2013). For instance, the P1 (Han et al., 1997, 1999, 2000; Heinze et al., 1998; Evans et al., 2000), the posterior N1 (Proverbio et al., 1998; Han et al., 2000; Beaucousin et al., 2011, 2013), the N2 (Han et al., 1997, 1999, 2000; Proverbio et al., 1998; Evans et al., 2000), and P3 (Han et al., 1997, 2000; Proverbio et al., 1998; Evans et al., 2000) components have all been found to be modulated when individuals focus their attention on either the global or local levels of Navon letters. However, there is not a clear consensus in whether viewing the global or local levels can systematically affect some ERPs and not others. For example, Heinze et al. (1998) and Evans et al. (2000) found that the attended level (i.e., global or local) influenced the amplitude of the P1 which was greater when attending globally vs. locally. In contrast, several studies by Han et al. (1997, 1999, 2000) observed that the P1 was larger when attending locally vs. globally. As well, several researchers have observed greater N1 amplitudes when attending to the global level than when attending to the local level (Proverbio et al., 1998; Han et al., 2000; Beaucousin et al., 2011, 2013), but others have not (Han et al., 1997, 1999; Evans et al., 2000). Differences between these results could be due to differences in the stimuli and/or tasks used. The processing of the global/local levels, as reflected in differences in ERP amplitudes, can be modulated by whether the stimuli presented in the global/local levels are congruent (e.g., the letter H composed of small H’s) or incongruent (e.g., the letter H composed of small T’s; Beaucousin et al., 2013), whether the task is a selective attention task (e.g., blocks of trials where individuals indicate targets in only the global or local levels) or divided attention task (e.g., trials where targets can be in either the local or global levels and individuals must attend to both; Heinze et al., 1998), stimulus factors that influence whether there is interference between one level relative to the other during processing (Proverbio et al., 1998; Beaucousin et al., 2013), and the complexity and density of the stimuli used (Leek et al., 2016).

Another potential modulator of ERPs when viewing global/local stimuli could be individual differences in the preference to attend to one level relative to the other. Results suggest that individuals differ naturally in the degree to which they are more inclined to focus on the global or local level, and individual differences in global/local biases have been shown to remain stable over a period of at least 10 days in multiple global/local tasks with a random student sample (Dale and Arnell, 2013). However, to date, there has not been systematic examination of whether individual differences in global/local bias are associated with individual differences in ERPs to the global/local stimuli. For example, will individuals with a global bias show amplitude differences in early and/or late ERPs relative to ERPs from individuals with a local bias? The only example we are aware of comes from Boksem et al. (2012). They found a greater positivity at approximately 300–400 ms when looking at a difference wave in ERPs at the C5 and C6 sites to global stimuli relative to local stimuli that predicted faster RTs to global stimuli. However, they did not control for local RTs, such as when a difference between local and global RTs is computed and used as a dependent measure, or when the RTs from one level are covaried out of the other level. Without knowing whether this difference wave was also associated with the difference in global and local RTs (or global RTs controlling for local RTs), this result does not provide evidence that individual differences in the ERP component are correlated with individual differences in global/local bias because global/local bias cannot be assessed using global RTs alone. By themselves, RTs to global stimuli reflect much more than global bias: for example, factors like motivation and general reaction time, processing speed, etc.

Although there has not yet been an examination of whether individual differences in RT differences when viewing global and local stimuli are associated with individual differences in ERPs to the global and local stimuli, some studies have examined groups that naturally differ in their attentional breadth and compared their ERPs while viewing the global/local stimuli. For example, individuals with schizophrenia have shown less global advantage in RTs relative to controls when viewing hierarchical stimuli (Dreben and Fryer, 1995; Landgraf et al., 2011). Schizotypal personality disorder (SPD) is a schizophrenia-related personality disorder that shares many common biological, genetic, and phenomenological characteristics with schizophrenia but the features of SPD (e.g., cognitive and social deficits) are often milder than schizophrenia (Siever and Davis, 2004). Choi et al. (2014) found faster RTs for global level targets relative to local level targets for controls, but no difference in global and local RTs for non-clinical college students who scored in the top 5% in their number of schizotypal features as measured by the Schizotypal Personality Questionnaire (SPQ). Choi et al. also showed that N1 amplitude (referred to as the subcomponent name of N150 by the authors) was significantly greater to local stimuli relative to global stimuli, but only for the high schizotypal trait group. In contrast, the P3 amplitude did not differ to local and global stimuli for the high schizotypal trait group but was greater to local stimuli relative to global stimuli for controls. Johnson et al. (2005) found that patients diagnosed with schizophrenia also showed slower responses to global stimuli relative to controls, and that these patients exhibited a larger N1 amplitude to local stimuli vs. global stimuli over the left hemisphere sites, and a reduced P3 amplitude to local stimuli relative to controls. Together, these results suggest that individual differences in attentional breadth may be associated with N1 and P3 amplitudes to global and local stimuli.

In the current study, ERPs were collected while participants performed a Navon letter task where participants reported which of two target letters was presented in an incongruent letter stimulus (see Figure 1). The target letter was presented unpredictably at the local or global level, with the other level displaying a non-target letter. The mean time was compared for global and local targets as a measure of attentional breadth. This design, where the participants must monitor both levels, allows us to better examine individuals’ natural attentional inclinations toward one level relative to the other in that their attention is not cued to either the global or local level. This target detection task with Navon letters has been used in other previous experiments to track individual differences in attentional breadth and to relate individual differences in attentional breadth with individual differences in neural measures such as ERPs and frontal alpha asymmetry (e.g., Harmon-Jones and Gable, 2009; Gable and Harmon-Jones, 2011; Pitchford and Arnell, 2019a,b). The target detection Navon task used here also allows us to estimate attentional breadth with a single difference score (local target RT − global target RT) as opposed to requiring a difference of the two difference scores that is needed for the original Navon version [(incongruent local − congruent local) − (incongruent global − congruent global)].

This Navon letters task was performed at two time points in the same testing session separated by a filler task of incongruent Stroop. Completing 5 min of incongruent Stroop has been previously shown to result in diverse changes in attentional breadth across individuals such that higher approach-motivated individuals showed narrowed attentional breadth following the completion of incongruent Stroop, whereas lower approach individuals showed broadened attentional breadth (Pitchford and Arnell, 2019b). Despite this finding, Pitchford and Arnell (2019b) showed no difference in attentional breadth in the sample overall, before completing incongruent Stroop vs. after incongruent Stroop, and no effects of performing congruent Stroop. These results suggest that completing incongruent Stroop can influence the breadth of attention but that attentional breadth changes after completing Stroop differ across individuals and that it may be the effortful nature of the incongruent Stroop task responsible for this diversity. Our goal was to have attentional breadth change as much as possible from Time 1 to Time 2 so that there would be enough variability in the dependent measure to track with ERPs. This allowed us to examine whether ERPs could predict why some individuals show narrowed attentional breadth at Time 2, whereas others show more broad attentional breadth at Time 2 to inform the neurocognitive processes that are involved in these changes in attentional breadth. It is much more effective and powerful to do this if there are large changes in attentional breadth than if there are smaller changes in attentional breadth and we hoped having the Stroop task in the middle of the two Navon blocks would result in greater diverse changes in attentional breadth than if there was nothing in the middle or a task that has not previously been shown to lead to diverse changes in attentional breadth. Further, greater changes in attentional breadth as computed by taking the difference between Time 2 and Time 1 allowed for a more powerful analysis when examining the relationship between ERPs and changes in attentional breadth in this study since examining the intra-individual differences in changes in attentional breadth from Time 1 to Time 2 allowed us to control for the individual differences in attentional breadth at Time 1.

We had two goals. The first goal was to examine whether interindividual differences in attentional breadth can be reflected in indicators of attentional engagement and processing during Navon letter trials *via* both earlier (P1 and N1) and later (P2, N2, and P3) ERPs. This is an important first step as there are no studies that have systematically examined whether interindividual differences in natural attentional breadth are reflected in interindividual differences in ERPs to global/local Navon stimuli. Based on the schizotypy/schizophrenia findings described above, we predict that individuals who are more focused on the local level, may show greater N1 and reduced P3 amplitudes when viewing the local level relative to the global level. However, it is possible that processing of Navon stimuli will not produce the same differences in ERPs as our sample consisted of a random student population.

The second goal of the current study was to examine whether intraindividual changes in the ERPs to hierarchical stimuli are associated with intraindividual changes in attentional breadth from Time 1 to Time 2 [i.e., either a shift to more narrowed (local) attentional breadth or a shift to more broad (global) attentional breadth from Time 1 to Time 2]. It may be that changes in ERPs to hierarchical stimuli from Time 1 to Time 2 are associated with changes in attentional breadth from Time 1 to Time 2. If changes in ERPs track with changes in breadth within individuals, then this could help us to better understand the mechanism that underlies the shift in attentional breadth over time while keeping factors like stimuli, task demands, and individuals constant to reduce the noise that could hide any signal. It is currently unclear whether ERP changes over time will track with changes in breadth over time. However, we hypothesize that changes in attentional breadth could potentially occur at the same stages of processing as above such that N1 and P3 amplitudes to Time 2 Navon stimuli relative to Time 1 Navon stimuli may relate to changes in attentional breadth.

## Materials and Methods

### Participants

Forty-two right-handed undergraduate students participated in this study for partial course credit (*Mage* = 20.86, *SDage* = 6.59; 36 females). The study received clearance from the Brock Research Ethics Board (REB clearance code 16–209) and all interactions followed approved procedures including obtaining informed consent from all participants. All participants in this study reported no history of neurological or cardiac conditions and were not taking psychoactive medications. They reported normal (or corrected-to-normal) vision and were able to discriminate colors.

### Procedure

The experiment took place in a dimly lit, shielded, sound attenuated room. Visual stimuli were presented on a 17-inch CRT monitor and controlled by E-prime software running on a Dell desktop computer. After completing informed consent, the electrode cap and electrodes were prepared. Participants then filled out questionnaires and completed an 8-min recording of resting electroencephalogram (EEG) – the results of which will not be discussed here. Participants then performed the first block of the Navon letter task. The participants were instructed to indicate which of two target letters had been presented (“H” or “T”) by pressing designated keys on the keyboard as quickly and accurately as possible. The design of the Navon task in the current study has been used extensively in past research to examine individual differences in attentional breadth and was chosen to track individual differences in attentional breadth (Harmon-Jones and Gable, 2009; Gable and Harmon-Jones, 2011; Gable et al., 2013; Threadgill and Gable, 2019). Target letters were shown randomly at the global or local level, so participants needed to be ready to attend to both levels on every trial. Participants completed 64 trials of the Navon task before performing 5 min of the incongruent Stroop task whereby they were presented with incongruent Stroop stimuli until 5 min had passed (Stroop, 1935). On each trial of the Stroop task, participants were instructed to indicate the font (i.e., ink) color of the incongruent word by pressing one of four keyboard keys with corresponding color stickers as quickly and accurately as they could. All trials were incongruent. Trials were completed for 5 min as a filler task. Following the Stroop task, the same 64 trials of the Navon task were again performed, but trials were shown in a different random order.

### Stimuli

Navon stimuli were large letter stimuli (global; visual angle of 3.82° by 2.39°) composed of smaller (local; visual angle of 0.19° by 0.19°) letter stimuli. Each stimulus was black and was presented centrally on a white-background screen. All hierarchical stimuli were incongruent where the identity of the global letter differed from the local letter (see Figure 1). On half of the trials, a target letter (H or T) was presented in the global level while a distractor letter (F or L) was presented in the local level. This was reversed for the other half of trials. All combinations of target letters, distractor letters, and target levels were equally likely and presented randomly trial-to-trial. On each trial, a fixation cross was presented for 500 ms, followed by a blank screen for 1,000 ms, and then a Navon letter which remained on the screen until a response was made indicating which of the two target letters had been presented. Each trial was followed by an inter trial interval of 1,000 ms.

### EEG Recording

EEG was recorded continuously using 29 tin electrodes embedded in an Electro-cap© (Electro-cap International Inc., Eaton, OH, United States) distributed according to the international 10–20 system. An electrode placed anterior to Fz was used as ground, while linked left and right earlobes were used as a reference. EEG data were amplified and acquired using a 32-channel NeuroScan SynAmps and Neuroscan acquisition software (Compumedics USA, Charlotte, NC, United States). EEG data were sampled online at a rate of 500 Hz. Electrooculogram (EOG) electrodes were placed on the outer canthus of each eye and on the infra‐ and supra-orbital regions of each eye to record horizontal and vertical eye movements, respectively. Impedance was maintained below 15 kohms. EEG data were analyzed offline using EEGLAB v14.1.1b (Delorme and Makeig, 2004) and custom routines written in MATLAB R2017a (The Mathworks, Natick, MA, United States). The data were band-pass filtered with the default EEGLAB filter (pop_eegfiltnew) excluding activity below 0.1 Hz and above 35 Hz and the default filter order (i.e., 2 Hz transition bandwidth). All activity +/−100 microvolts in the vertical electrooculogram and horizontal electrooculogram was rejected from analysis. Afterward, epochs with artifacts (i.e., signals due to muscle movement, eye movements, and eye blinks) were manually removed using visual inspection and rejected from analysis. Epochs were created that began 200 ms prior to Navon Letter presentation and ended 500 ms after Navon Letter presentation, for each of the four conditions (Time 1 global target, Time 1 local target, Time 2 global target, and Time 2 local target; mean number of trials accepted for analysis per participant were 23, 24, 23, and 22, respectively). Time windows for early ERP components (P1: 80–130; N1: 130–200 ms) and later ERP components (P2: 210–240; N2: 240–270; and P3: 270–490 ms) were chosen based on a combination of viewing the grand averaged mean and time windows used in previous research (Proverbio et al., 1998; Garrison et al., 2017). ERPLAB (Lopez-Calderon and Luck, 2014) allowed for epochs to be averaged within each of the conditions (Time 1 local, Time 1 global, Time 2 local, and Time 2 global) for each individual. We were mainly interested in examining ERPs related with attentional and perceptual processing and which have been examined in previous research with global/local processing, so we focused on sites in the parieto-occipital region (P3, PZ, P4, O1, OZ, and O2) since the effects of global/local processing on ERPs have been consistently found using those sites (Johnson et al., 2005; Beaucousin et al., 2011, 2013; Leek et al., 2016). Similar to other research (Heinze et al., 1998; Proverbio et al., 1998; Evans et al., 2000; Yamaguchi et al., 2000), the ERPs recorded within the group of sites were averaged for the statistical analyses.

### Data Analysis

To determine whether individual differences in ERPs to global and local stimuli predicted individual differences in attentional breadth (see Table 1 for means and SDs), we conducted multiple regressions for each of the components of interest (P1, N1, P2, N2, and P3) where the average component to local stimuli was entered in the same step of the model as the average component to global stimuli (see Table 2). By entering both the local and global predictors into the model at the same time, the variability common to both was removed from each and we were able to examine residual measures of global and local ERPs which included only variability in the average amplitude of an ERP unique to that one level of the hierarchical stimuli (i.e., variability in the P3 to the global targets that was not shared by local targets). In this way, variability due to confounds such as general processing speed, was excluded. This approach allowed us to examine the unique contribution of each component to global and local stimuli separately in explaining individual differences in attentional breadth, whereas this would not be possible when using differences in ERP amplitude when viewing global and local stimuli. Conducting the multiple regression analyses allowed us to then extract residualized ERP measures (e.g., P3 amplitude to local stimuli while controlling for P3 amplitude to global stimuli) to produce scatterplots showing the relationships between the residualized ERP measures and attentional breadth.

**Table 1:** Descriptive statistics for attentional breadth measures (ms) at Time 1 and Time 2, as well as the change in attentional breadth from Time 1 to Time 2 (*n* = 42).

	Mean	SD	Minimum	Maximum
Time 1 local RT	732	141	515	1,037
Time 1 global RT	700	134	462	1,077
Time 2 local RT	632	119	434	942
Time 2 global RT	612	102	423	924
Time 1 breadth	31	106	−196	288
Time 2 breadth	21	76	−151	196
Change in breadth	−11	86	−201	154

Breadth is calculated as local RT − global RT.

**Table 2:** Simultaneous regression analysis of Time 1 attentional breadth as predicted by mean ERP amplitudes to Time 1 global and local stimuli.

Predictor	*R^2^*	*SPr*	*β*	*t*	*p*
P1	0.01				
Local		<0.001	<0.001	<0.001	0.99
Global		−0.09	−0.11	−0.54	0.59
N1	0.07				
Local		0.27	0.37	1.73	0.09
Global		−0.18	−0.25	1.16	0.25
P2	0.08				
Local		0.24	0.31	1.59	0.12
Global		−0.25	−0.32	1.66	0.11
N2	0.08				
Local		0.21	0.29	1.38	0.18
Global		−0.28	−0.39	1.83	0.07
P3	0.04				
Local		0.13	0.23	0.89	0.38
Global		−0.03	−0.06	−0.21	0.83

SP, semi-partial. Dependent variable: Time 1 attentional breadth. *p < .05

To determine whether changes in attentional breadth from Time 1 to Time 2 (attentional breadth at Time 2 minus attentional breadth at Time 1 where lower numbers represented a shift toward more narrowed attentional breadth) could be tracked by changes in ERPs from Time 1 to Time 2, hierarchical regressions were conducted for each of the separate ERP components. In the first step, changes in attentional breadth were regressed on the mean amplitudes for Time 1 global and local Navon stimuli for all components of interest. Examining the results from Step 1 could then tell us whether the change in attentional breadth was predicted by ERPs to hierarchical stimuli at Time 1. In the second step, for each ERP component model, we then regressed changes in attentional breadth on the mean amplitudes for Time 2 global and local Navon stimuli while controlling for the Time 1 ERPs entered in the first step (see Table 3). This allowed us to examine whether the change in attentional breadth was predicted by ERP amplitude at Time 2 over and above the ERP amplitude at Time 1 for each component.

**Table 3:** Hierarchical regression analysis of changes in attentional breadth as predicted by mean ERP amplitudes to Time 1 global and local stimuli (step 1), and Time 2 global and local stimuli, while controlling for predictors in step 1 (step 2).

Predictor	Step 1 (Time 1)				Step 2 (Time 2)			
	*R^2^*	*SPr*	*β*	*p*	*ΔR^2^*	*SPr*	*β*	*p*
P1	0.04				0.13			
Local		0.15	0.20	0.33		−0.13	−0.17	0.40
Global		−0.01	−0.01	0.97		0.35	0.48	0.02
N1	0.03				0.19^*^			
Local		−0.11	−0.14	0.51		−0.40	−0.74	0.01
Global		0.16	0.23	0.30		0.33	0.63	0.03
P2	0.02				0.07			
Local		0.00	0.00	0.99		−0.26	−0.46	0.11
Global		0.11	0.13	0.50		0.17	0.29	0.29
N2	0.05				0.07			
Local		−0.01	−0.02	0.94		−0.24	−0.44	0.13
Global		0.17	0.23	0.29		0.01	0.01	0.98
P3	0.02				0.16^*^			
Local		−0.12	−0.19	0.47		−0.38	−0.61	0.02
Global		0.13	0.21	0.42		0.24	0.44	0.12

*N* = 42. SP, semi-partial. Dependent variable: change in attentional breadth.

* p < 0.05.

## Results

### Navon Letter Task

All correct global/local RTs that were less than 2,000 ms were extracted (*M*reject = 2.68%, *SD*reject = 1.81%). These were subjected to a two-stage recursive outlier elimination procedure where RTs were removed if they were greater or less than 2 SDs from the mean for each combination of participant, level (global and local) and time (Time 1 Navon and Time 2 Navon; *M*reject = 8.80%, *SD*reject = 1.81%).

Similar to previous research, a difference measure was calculated (local RT − global RT), to represent attentional breadth (e.g., Juergensen and Demaree, 2015; Pitchford and Arnell, 2019a,b). Attentional breadth change values were calculated as Time 2 Navon attentional breadth minus Time 1 Navon attentional breadth, such that values greater than 0 represented a shift toward broader attention at Time 2, and values less than 0 represented a shift toward more narrowed attention at Time 2, relative to Time 1.

There was a significant positive relationship between attentional breadth at Time 1 and Time 2, *r* = 0.60, *p* < 0.001, which is consistent with previous research (e.g., Dale and Arnell, 2013), suggesting that there are reliable individual differences in global/local bias (see Table 1 for means and SDs). Overall, attentional breadth did not differ for the Time 1 Navon block relative to the Time 2 Navon block, *t*(41) = 0.83, *p* = 0.41. Reaction times for hierarchical stimuli presented at Time 1 and Time 2 were also highly correlated (local RTs: *r* = 0.71, global RTs: *r* = 0.72). Reaction times for hierarchical stimuli as well as attentional breadth at Time 1 and Time 2 were associated with reaction times to Stroop stimuli such that faster Stroop RTs were associated with narrower attentional breadth both before and after completing Stroop breadth (*r*’s were 0.34 and 0.43 for Time 1 and Time 2). This suggests that a greater local focus might have aided individuals in completing the Stroop task.

### ERP Results

**Figure 2:**
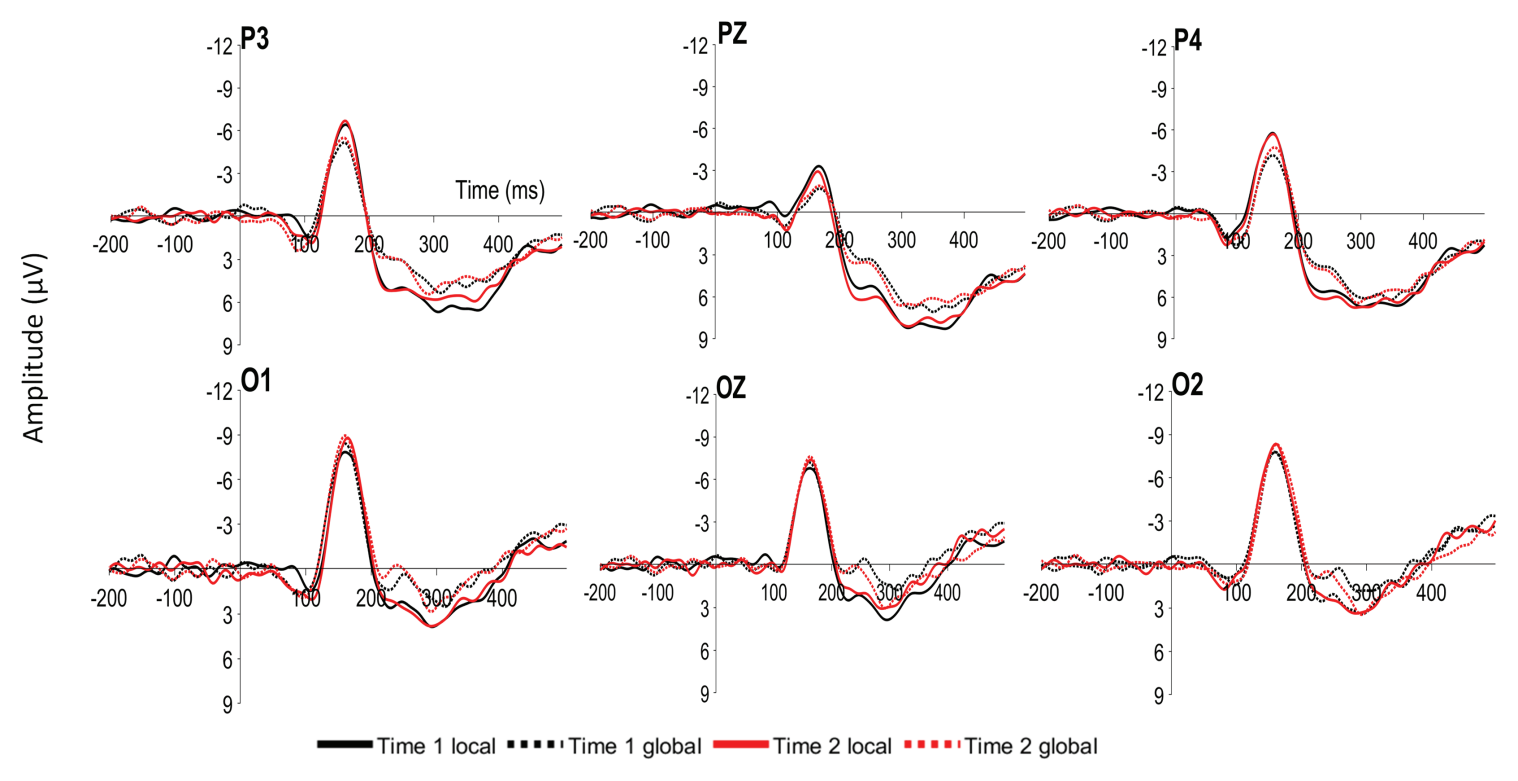
Grand average event-related potentials (ERPs) at parietal (P3, PZ, and P4) and occipital (O1, OZ, and O2) sites time locked to local and global targets presented at Time 1 (Time 1 local and Time 1 global) and Time 2 (Time 2 local and Time 2 global).

**Figure 3:**
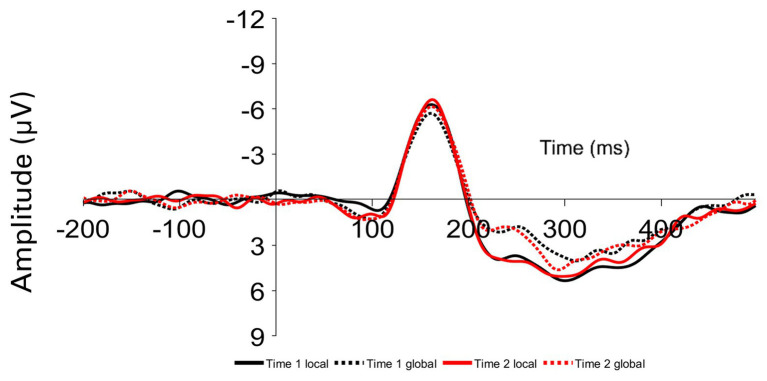
Grand average ERPs averaged over parietal and occipital sites time locked to local and global targets presented at Time 1 (Time 1 local and Time 1 global) and Time 2 (Time 2 local and Time 2 global).

Participants viewed both global and local target Navon stimuli at Time 1 and Time 2, resulting in four within-subject-conditions: Time 1 Navon when the target was in the local level, Time 1 Navon when the target was in the global level, Time 2 Navon when the target was in the local level, and Time 2 Navon when the target was in the global level. The mean amplitudes of the P1, N1, N2, P2, and P3 components were examined at occipital and parietal sites (O1, O2, OZ, P3, P4, and PZ; see Figure 2). Given that the wave patterns were similar at all six sites, these six sites were averaged together (Figure 3). Mean amplitudes to global and local stimuli were highly correlated for each ERP component and time point, *r*’s ranging from 0.60 to 0.80, *p*’s < 0.001.

To determine whether ERP amplitudes differed by the level and Time 1/Time 2 conditions, a 2 (Time 1 Navon/Time 2 Navon) × 2 (local level/global level) within-factors ANOVA was conducted for each of the P1, N1, P2, N2, and P3 components. The two-way interaction was not significant for any of the components, *p*’s > 0.28. As seen in Figure 3, there is a clear difference in the amplitudes of global and local stimuli from approximately 200–300 ms, whereby there were significantly greater P2 amplitudes, *F*(1,41) = 16.84, *p* < 0.001, *η*^2^_p_ = 0.29, and significantly lesser N2 amplitudes, *F*(1,41) = 15.00, *p* < 0.001, *η*^2^_p_ = 0.27 to local stimuli relative to global stimuli. Mean amplitudes did not differ between the two time points for any of the components, *p*’s > 0.28.

### Relationships Between Attentional Breadth and ERPs to Global/Local Stimuli

To examine whether interindividual differences in natural attentional breadth were reflected in interindividual differences in ERPs to global/local Navon stimuli, mean amplitudes to both local and global stimuli presented in the first Navon block were used as predictors in a regression to determine whether they predicted attentional breadth in the first Navon block. Overall, none of the regression models predicted a significant amount of variability in interindividual attentional breadth differences, *p*’s > 0.19. There were a few trends for specific predictors but mean residual amplitudes of ERP components did not significantly correlate with attentional breadth, *p*’s > 0.07. Therefore, interindividual differences in ERP amplitudes from Time 1 Navon trials were not significantly related to interindividual differences in attentional breadth from Time 1 Navon trials. Latencies for each of the components in response to global and local stimuli presented at Time 1 and Time 2 were also examined. There was a significant relationship whereby shorter N2 latencies to Time 2 local stimuli predicted narrowed attentional breadth at Time 2, *r* = 0.44, *p* < 0.01. None of the other latency ERP measures were convincingly associated with any of the attentional breadth measures.

The second goal was to examine whether the changes in electrophysiological activity to hierarchical stimuli presented from Time 1 to Time 2 could predict changes in attentional breadth from Time 1 to Time 2. The overall models for the components to Time 1 global/local stimuli did not explain a significant amount of variance in the change in attentional breadth, *R*^2^ values ranging from 0.02 to 0.05, *p*’s > 0.38. Further, none of the ERP components to either global or local stimuli presented at Time 1 could explain a significant amount of unique variability in the change in attentional breadth, *p*’s > 0.29. Therefore, individual differences in ERP amplitudes from the Time 1 block of Navon trials were not significantly related to individual differences in attentional breadth changes from Time 1 to Time 2. We later conducted the same analyses separately for ERPs to local stimuli only, or global stimuli only. We did not find any significant relationships when examining P3, N1, P2, or N2 ERPs to only one level and the change in attentional breadth, although we did find that the Time 2 global P1 amplitude was a unique significant predictor of the change in attentional breadth when controlling for Time 1 global P1 amplitude, semi-partial *r* = 0.35, *p* = 0.03.

**Figure 4:**
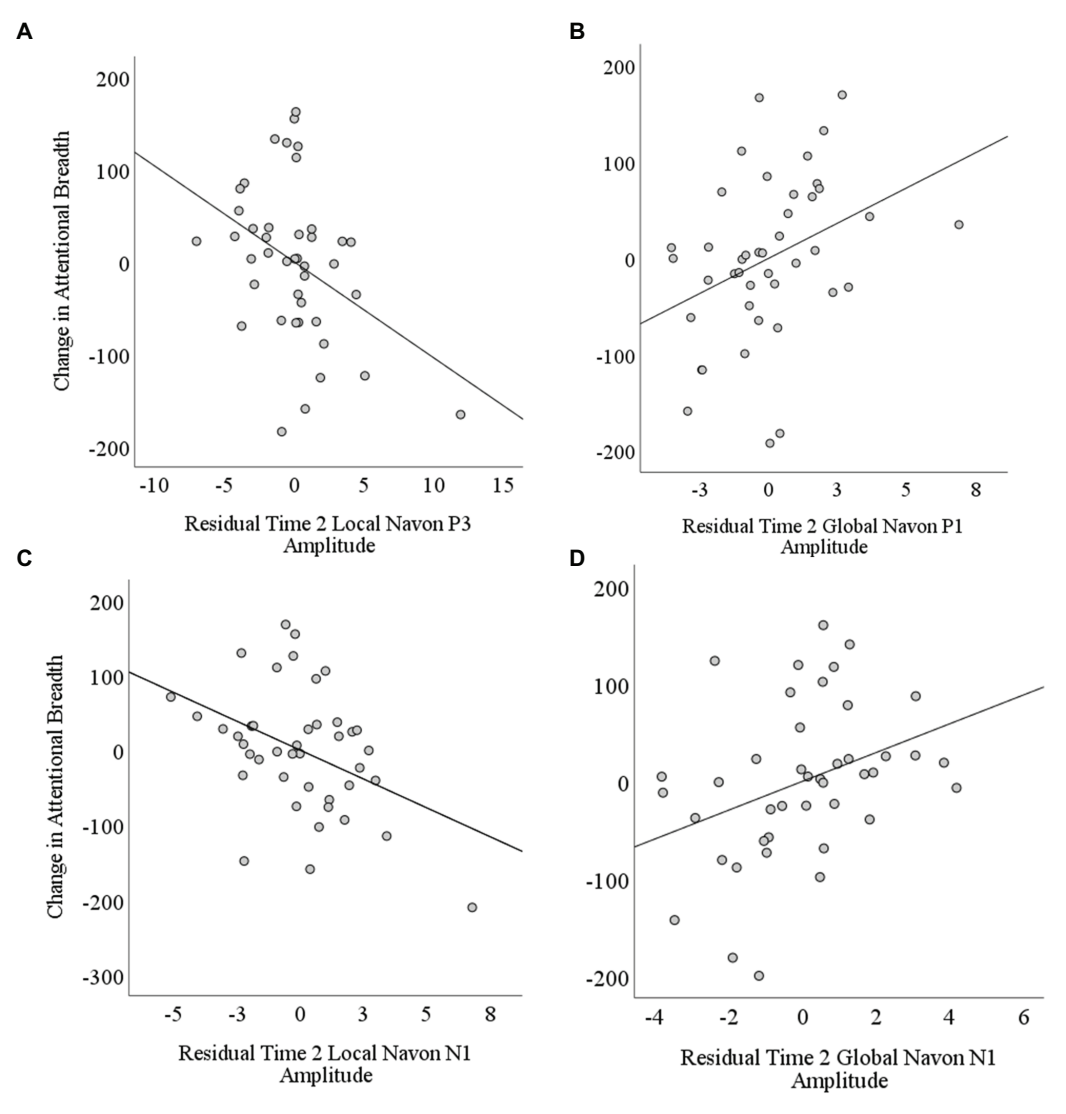
Scatterplots depicting correlations between changes in attentional breadth and residual Time 2 amplitudes controlling for Time 1 amplitudes as a function of stimulus level (local and global) and component (P1, N1, and P3). The change in attentional breadth was significantly predicted by Time 2 residual (A) P3 amplitude to local level stimuli, (B) P1 amplitude to global level stimuli, (C) N1 amplitude to local level stimuli, and (D) N1 amplitude to global level stimuli.

In the second step, for each ERP component model, we then regressed changes in attentional breadth on the mean amplitudes for Time 2 global and local Navon stimuli while controlling for the Time 1 ERPs entered in the first step (see Table 3). When changes in attentional breadth were regressed on P1 amplitudes to Time 2 local and global Navon letters, they explained a nonsignificant 13% of variance over and above the P1 amplitudes to Time 1 hierarchical stimuli, Δ*R*^2^ = 0.13, *F*(2,37) = 2.77, *p* = 0.07. Mean residual P1 amplitude to Time 2 local Navon letters was not a significant, unique predictor of changes in attentional breadth, semi-partial *r* = −0.13, *p* = 0.40 but mean P1 residual amplitude to Time 2 global Navon letters was a significant, unique predictor of changes in attentional breadth, semi-partial *r* = 0.35, *p* = 0.02 (see Table 3; Figure 4A), such that greater residual P1 amplitude to Time 2 global Navon letters was associated with a shift toward increased attentional breadth.

When changes in attentional breadth were regressed on N1 amplitudes to Time 2 local and global Navon letters in step 2, they explained a significant 19% of variance over and above the N1 amplitudes to Time 1 hierarchical stimuli, Δ*R*^2^ = 0.19, *p* = 0.02 (see Table 3). Mean N1 residual amplitude to Time 2 local Navon letters, semi-partial *r* = −0.40, *p* = 0.01 (see Figure 4C), and N1 amplitude to Time 2 global Navon letters, semi-partial *r* = 0.33, *p* = 0.03 (see Figure 4D), were each significant, unique predictors of changes in attentional breadth such that lesser N1 residual amplitude to Time 2 local stimuli was associated with a shift toward more narrowed attentional breadth at Time 2, while lesser N1 residual amplitude to Time 2 global Navon stimuli was associated with a shift toward increased attentional breadth.

When changes in attentional breadth were regressed on P3 amplitudes to Time 2 local and global Navon letters in the second step, they explained a significant 16% of variance over and above the P3 amplitudes to Time 1 hierarchical stimuli, Δ*R*^2^ = 0.16, *p* = 0.04 (see Table 3). Residual P3 amplitude to Time 2 local Navon letters, semi-partial *r* = −0.38, *p* = 0.02 (see Figure 4B), was a significant, unique predictor of changes in attentional breadth such that greater residual P3 amplitude to Time 2 local Navon Stimuli was associated with a shift toward more narrowed attentional breadth at Time 2. Mean P3 residual amplitude to Time 2 global Navon letters was not a significant, unique predictor of changes in attentional breadth, semi-partial *r* = 0.24, *p* = 0.12.

When changes in attentional breadth were regressed on P2 amplitudes or N2 amplitudes to Time 2 local and global Navon letters, after controlling for amplitudes to Time 1 hierarchical stimuli, neither predicted significant variability above and beyond the variability explained in step 1, P2: Δ*R*^2^ = 0.07, *p* = 0.27, ΔN2: *R*^2^ = 0.07, *p* = 0.27 (see Table 3). Residual P2 and N2 amplitudes to global and local Navon letters presented at Time 2 were not significant unique predictors of changes in attentional breadth, *p*’s > 0.11.

The pattern of results reported in Table 3 remained the same when only step 2 of the model was included for each component, with the exception of the P3 component where both the Time 2 global and local P3 amplitudes were now significant unique predictors of changes in attentional breadth when not controlling for Time 1 P3 amplitudes. The pattern of results for the N1 and P3 components were similar when examining each of the six electrode site separately with the exception that the pattern was not significant at the O2 site for the N1 component or the P3 site for the P3 component. The pattern of results for the P1 component was prevalent across all electrode sites but was only significant when examining either the O1 and OZ sites on their own. Further, we did not see any evidence when looking at the pattern of results at separate sites to suggest that there was a difference between left and right electrodes or parietal and occipital electrodes except for the P1 component where the pattern was only significant at the mid-to-left electrode sites when analyzing each site separately. To conclude, the pattern of results reported here is seen throughout the parietal and occipital sites for the N1 and P3 components but was most strongly seen in the middle-to-left occipital sites for the P1 component.

Together, these results suggest that larger residual P1, and smaller residual N1 amplitudes to global stimuli were associated with a shift toward more broad attention following from Time 1 to Time 2, while larger residual P3s and smaller N1s to local stimuli were associated with a shift toward more narrowed attentional breadth from Time 1 to Time 2. It should be noted that the correlations between the significant, unique N1 and P3 predictors were fairly high, *r*’s ranging from 0.49 to 0.80, whereas there was a rather small association between the P1 amplitude to global stimuli at Time 2 and the significant, unique N1 and P3 predictors, *r*’s ranging from −0.09 to 0.16.

## Discussion

There were two goals for this study. The first goal was to examine whether interindividual differences in attentional breadth were reflected in indicators of attentional engagement and processing during Navon letter trials *via* both earlier (P1 and N1) and later (P2, N2, and P3) ERPs. There were a few trends such that greater N1 amplitude to local stimuli was associated with local attentional bias, while greater N2 amplitude to global stimuli was associated with global attentional bias, but neither relationship was significant. If there are differences in processing between individuals that show a local bias relative to those that show a global bias, it most likely occurs in the middle stages of processing. This is shown by the greater variability explained by N1, N2, and P2 amplitudes relative to P1 and P3 amplitudes when explaining individual differences in attentional breadth. However, overall, the results presented here suggest that individual differences in attentional breadth are not robustly reflected in ERPs to global/local stimuli.

The second goal was to examine whether the changes in electrophysiological responses to hierarchical stimuli at a second time point, relative to a first time point, were associated with changes in attentional breadth [i.e., either a shift to more narrowed (local) attentional breadth or a shift to more broad (global) attentional breadth from Time 1 to Time 2]. If changes in ERPs track with changes in breadth within individuals, then this could help us to better understand the mechanism that underlies the shift in attentional breadth over time while keeping stimuli, task demands, and individuals constant, even if individual differences in ERPs to global/local stimuli are not associated with individual differences in reaction times to those stimuli. Changes in attentional breadth could be partially predicted by changes in N1 and P3 amplitudes to Navon stimuli such that lesser N1 amplitude to the Time 2 global stimulus was associated with a shift toward increased attentional breadth, while greater P3 and lesser N1 amplitudes to the Time 2 local stimulus were associated with a shift toward narrowed attentional breadth. Greater P1 amplitude to Time 2 global stimuli was also associated with a shift toward increased attentional breadth. In contrast, for these same components, Time 1 amplitudes to local and global stimuli did not associate with changes in attentional breadth.

It is unclear why the P1 amplitude increases at Time 2 to global stimuli was associated with a shift toward more broad attentional breadth from Time 1 to Time 2. We can largely rule out differences in sensory information since the stimuli remained the same at both time points. One possibility is that individuals who switched their attentional focus more toward the global level of the stimuli may have increased their attention on that level, and thus a greater P1 may simply reflect the greater attention on that level. Another possibility is that a decreased N1 is more positive, and some of this positivity may have fallen into the late P1 window, although we did not find a strong correlation between the two ERP amplitudes.

Why might N1 amplitudes be smaller for stimuli that matched the direction of the breadth change (local for narrowed breadth and global for increased breadth)? One possibility is that participants shift their attention toward their preferred level. Once their attention is more focused on that level, the N1 may reflect the difference between when the target is presented in their preferred level and the added discrimination needed when the target is not presented in the preferred level. For example, if the target is presented locally and one’s attention is more focused on the smaller letters in the Navon level, they do not need to discriminate the letter at the other (global) level to correctly identify the target letter. In contrast, if the target is presented in their non-preferred level, then they now must discriminate and attend to the other level also. Past literature has shown that the N1 is larger when making voluntary discrimination processes relative to simple detection (Vogel and Luck, 2000; Hopf et al., 2002). This also suggests that the relationship between changes in attentional breadth and N1 amplitudes may be different when examining global/local attention during a blocked Navon letter task where participants are cued to attend to the global level (or local level) or an entire block of trials given that there would be less ambiguity in terms of where the target letter would be. The smaller N1 amplitude when the target is in the newly prioritized level, as seen by the shift in attentional breadth toward that level, might suggest lesser discrimination is needed than when the target is in the less prioritized level.

Overall, we found that there was a greater positivity at approximately 200 ms, suggesting a greater P2 and lesser N2 when viewing local level stimuli relative to global level stimuli. This pattern of results partially matches the results by Proverbio et al. (1998) in that they also showed a greater negative deflection at approximately 200 ms for global relative to local stimuli, but other work by Han et al. (1997, 1999) and Evans et al. (2000) found the opposite pattern where the N2 was greater when participants attended to the local level relative to the global level. Although there was a difference in N2 and P2 amplitude when viewing global vs. local stimuli, there was no evidence to suggest that the difference in N2 or P2 amplitude between the two levels related with individual differences in attentional breadth or the change in attentional breadth from Time 1 to Time 2.

P3 amplitudes were larger for stimuli that matched the direction of the breadth change. The P3 component reflects processes underlying stimulus evaluation, categorization, and encoding into working memory (e.g., Donchin, 1981). The P3 amplitude has been associated with attentional selection, and reductions in P3 amplitude predict attention control errors (e.g., Ritter et al., 1972; Datta et al., 2007). The P3 is larger for stimuli that are noteworthy such as rare stimuli (Squires et al., 1975). The larger P3 for stimuli that matched the direction of the breadth change is consistent with the idea that the preferred level would receive more attention, and be preferentially selected and encoded over the less preferred level.

One might reasonably ask whether the incongruent Stroop task was required between the two Navon blocks, and whether individual changes in breadth were due to completing the Stroop task, or if the same pattern of results could be observed by simply having participants perform the Navon letter task twice separated by a few minutes. It is impossible to know the role of the incongruent Stroop task here, as there is no condition where participants did not perform incongruent Stroop as the filler task between the two Navon blocks. However, for our purposes here, it does not matter whether or not individual differences in attentional breadth resulted from the incongruent Stroop task *per se*. Here, we wished to examine whether ERPs to hierarchical letters were associated with attentional breadth and/or changes in attentional breadth. Pitchford and Arnell (2019b) showed that the incongruent Stroop task specifically could modulate attentional breadth differently for different individuals, whereby there was an interaction between the congruency of the Stroop task completed and individual differences in approach-motivation where attentional breadth narrowed for higher approach individuals after completing incongruent Stroop but broadened for lower approach-motivated individuals, and this pattern only applied to individuals that had completed incongruent Stroop and not congruent Stroop. Given that we wanted to predict attentional breadth and changes in attentional breadth, the use of incongruent Stroop as a filler task seemed likely to heighten the chances that attentional breadth would be modulated over time, and that this modulation would not be uniform across participants. Therefore, the incongruent Stroop task was a vehicle to produce attentional breadth changes that could be associated with ERPs. Indeed, although participants did not become more locally or globally biased as a group (a non-significant 11 ms difference in breadth from Time 1 to Time 2 here replicated; Pitchford and Arnell, 2019b), there were large individual differences in attentional breadth changes over time (from 201 ms more local to 154 ms more global at Time 2 compared to Time 1), and these were reflected in N1 and P3 differences.

Although the sample size for this experiment was large relative to most others examining ERPs, a *post hoc* power analysis was conducted in G*Power (Faul et al., 2007) and showed that there was sufficient power to find moderate-to-large individual difference relationships [*R*^2^ = 0.17, power (1 − *β*) = 0.80] between our measures in the current experiment. One explanation as to why we found that ERPs predicted changes in breadth but did not predict attentional breadth during Time 1 might be because changes in attentional breadth reflected changes within each individual (intra-individual differences), whereas the variability in attentional breadth at Time 1 encompassed only inter-individual differences in attentional breadth. Although both inter‐ and intra-individual differences approaches are valid, examination of intra-individual differences is potentially more powerful given the numerous extraneous factors that are controlled for a within-participant design relative to a between-participant design (assuming meaningful changes in breadth within individuals). The results presented here suggest that the inter-individual differences in attentional breadth may not relate with ERPs to hierarchical stimuli but it is possible that these relationships were small and we did not have enough power in the current experiment to find these smaller effects. Indeed, P1, N2, and P2 components had relationships of 0.21 or better, suggesting the need for a more powerful future experiment on this question.

This is the first study to examine whether ERPs to global/local stimuli were associated with interindividual differences in attentional breadth, as well as whether changes in ERPs to global/local stimuli are associated with changes in attentional breadth within an individual over time. The evidence presented here did not support our hypothesis that interindividual differences in attentional breadth could be reflected in interindividual differences in ERPs to global/local Navon letter stimuli. However, there was evidence to support our hypothesis that changes in attentional breadth were related to changes in electrophysiological responses when viewing Navon letters over two different time points. The findings from this study suggest that changes in attentional breadth are reflected in individual differences in the P1, N1, and P3 components at Time 2 such that smaller N1s and larger P3s accompany a shift to processing the newly prioritized level while greater P1s to global stimuli predict a shift toward greater breadth. This suggests that both perceptual and later attentional processes are involved in the shifting of attentional breadth, whereby lesser discrimination and perceptual processing and greater attentional allocation are elicited when viewing the newly preferred level of the hierarchical stimuli.

## Data Availability Statement

The raw data supporting the conclusions of this article will be made available by the authors, without undue reservation.

## Ethics Statement

The studies involving human participants were reviewed and approved by Brock University Research Ethics Board. The patients/participants provided their written informed consent to participate in this study.

## Author Contributions

BP and KA designed and executed the experiment. BP analyzed the data and wrote the manuscript with edits from KA. Both authors read and approved the final manuscript.

### Conflict of Interest

The authors declare that the research was conducted in the absence of any commercial or financial relationships that could be construed as a potential conflict of interest.
